# Application of multi-omics analysis in atherosclerosis

**DOI:** 10.1186/s13019-026-04648-y

**Published:** 2026-07-29

**Authors:** Meiling Jiang, Xu Xu, Guofu Zhu

**Affiliations:** 1 Cardiology department, the Second Affiliated Hospital of Kunming Medical University, Kunming, 650000 Yunnan Province China; 2 General Medicine Department, affiliated Calmette Hospital of Kunming Medical University, Kunming, 650000 Yunnan Province China

**Keywords:** Atherosclerosis, Multi-omics analysis, Pathogenesis, Early diagnosis and treatment, Risk prediction

## Abstract

Atherosclerosis is a leading global cause of disability and mortality, with a rising trend of early onset across populations. Its pathogenesis is multifactorial, encompassing multiple interconnected pathways including dyslipidemia, inflammation, and immune dysregulation. Current therapeutic strategies targeting lipoproteins are insufficient to address disease progression, and there remains an urgent unmet clinical need to further elucidate its pathogenic mechanisms, refine diagnostic approaches, and identify novel therapeutic targets. In recent years, the vigorous development of omics technology has provided help for in-depth investigation of the pathogenesis of diseases and search for new diagnosis and treatment targets. The application of multi-omics analysis has elevated atherosclerotic research to a new tier, yielding high-quality findings that further unravel atherogenic molecular mechanisms. These findings provide critical theoretical and translational groundwork for developing novel diagnostics, targeted therapies, and robust early risk stratification tools. In this review, we summarize integrated multi-omics applications in atherosclerosis, and outline novel perspectives for prospective therapeutic strategies.

**Supplementary Information:**

The online version contains supplementary material available at https://doi.org/10.1186/s13019-026-04648-y.

## Introduction

Atherosclerotic cardiovascular diseases(ASCVD) is the leading global cause of mortality [1]. Atherosclerosis (AS) is a lipid storage disorder arising from intimal arterial lipoprotein (especially LDL) accumulation, which are modified and phagocytosed by scavenger receptor‑mediated inflammatory macrophages to form plaques [2]. Even with effective conventional interventions targeting LDL, blood pressure and other traditional risk factors, therapeutic efficacy remains suboptimal, leaving substantial residual ASCVD risk [3].

Omics technologies aim to decode molecular mechanisms across genomics, proteomics and metabolomics to identify novel disease mechanisms and biomarkers [4]. Technical advances enable heterogeneity analysis of anatomically diseased vessels and associated atherosclerotic plaques, revealing insights into the microenvironment, cellular evolutionary trajectories and immune response pathways [5]. Progress in next-generation sequencing, high-resolution mass spectrometry, multi-omics integration methods and comprehensive databases has driven omics research from traditional single-omic approaches to more sophisticated multi-omics frameworks [6]. Multi-omics integration is an emerging holistic strategy to comprehensively characterize molecular and physiological events underlying human diseases, including dyslipidemia, AS, and inflammation [7]. It can be utilized not only for risk prediction and early diagnosis but also as a guide for personalized dietary therapies and prognostic regimens. This approach provides fresh insights into the mechanisms and pathways that link genotypes with clinically relevant traits as well as treatment outcomes [8]. Next, we will present the application of multi-omics analysis integrating various omics methods in AS mechanism research, treatment strategies, and risk prediction. This emerging approach plays an important role in advancing the development of AS research.

## Transitioning from single-omics to multi-omics

Omics technologies encompass a diverse array of fields, including genomics, epigenomics, metagenomics, transcriptomics (examining RNA expression patterns), proteomics, and metabolomics. These technologies also interact with dietary and nutritional factors [7]. Genomics is the comprehensive study of the structure, function, and evolution of an organism’s genome [9]. Recent advancements stemming from the Human Genome Project have significantly enhanced our understanding of the genetic foundations underlying complex traits such as AS [10]. n transcriptomics, mRNA expression levels are tightly correlated with AS disease status, and targeted modulation of specific mRNAs has demonstrated efficacy in regulating inflammation in advanced atherosclerotic lesions, representing a promising therapeutic strategy for AS [11]. Distinct expression patterns of non-coding RNAs (ncRNAs) — including long non-coding RNAs (lncRNAs), microRNAs (miRNAs), circular RNAs (circRNAs), and Piwi-interacting RNAs (piRNAs) — have been consistently identified in atherosclerotic vascular diseases [12, 13]. Proteomic analysis of atherosclerotic plaques enables redefinition of clinically relevant patient cohorts based on differential prognostic outcomes, and facilitates identification of subgroups of male and female patients at elevated risk for future cardiovascular events [14, 15]. Epigenetic studies support the classification of AS as an epigenetic disorder driven by the interplay of three core interrelated epigenetic processes: DNA methylation, histone methylation/acetylation, and regulatory ncRNAs, all of which play critical roles in atherosclerotic plaque development and destabilization [16, 17]. Aberrant methylation at specific genomic sites has also been firmly associated with AS pathogenesis [18]. In metabolomics, plasma metabolomic analysis is critical for characterizing metabolic disorders, and alterations in small molecule metabolites can serve as indicators of underlying coronary artery disease (CAD) as well as biomarkers for CAD progression [19, 20]. Microbiome research has confirmed links between the microbiome, microbiota-derived compounds, and AS [21, 22]. Specific gut microbiota-associated metabolites including trimethylamine-N-oxide (TMAO), short-chain fatty acids (SCFAs), and bile acids (BAs) are also correlated with the onset, prevention, treatment, and prognosis of cardiovascular diseases [23]. The summary of single-omics data can be found in Supplementary Table 1.

Single omics approaches exhibit notable limitations, as they can only be examined from a singular perspective and fail to elucidate the overall functionality of the organism or clarify the interplay between biological and environmental factors [24]. Transcriptomics detects up/downregulation of core biosynthetic and signaling genes, yet genetic alterations do not always directly correlate with protein abundance changes; proteomics identifies cellular/tissue enzyme and PTM alterations but cannot pinpoint which enzymes are actively generating metabolites in pathological states; metabolomics effectively identifies bioactive signaling molecules, while therapeutic interventions require well-defined protein targets [25]. Multi-omics integration leverages cross-layer data alignment and causal inference frameworks to identify novel disease causal mediators undetectable by single-omics analyses. In atherosclerotic (AS) research, it employs standardized workflows including cis-regulatory QTL colocalization, mediation analysis, and two-sample Mendelian randomization (MR) to disentangle upstream genetic perturbations from downstream transcriptomic, proteomic and metabolic alterations, distinguishing true mechanistic causal links from spurious statistical associations [26–28].

## Application of machine learning to integrate multi-omics data in atherosclerosis

The accumulation of multi-omics data offers a valuable resource for investigating this condition [29]. “Multi-omics” data are now integrated into several international databases such as the Human Microbiome Project (HMP), the Human Proteome Project (HPP), and the Human Metabolome Database (HMDB) [30]. These comprehensive publicly available datasets facilitate research on disease-related tissues across various biological levels of multi-omics analysis while offering clear insights into the relationships between risk factors and diseases [31]. However, the high dimensionality, heterogeneity, and complexity inherent in multi-omics data pose significant challenges to traditional analytical methods.

Machine Learning (ML) serves as a powerful data analysis tool that can extract valuable insights from vast datasets. It offers innovative solutions for mechanism research, risk prediction, and personalized treatment of AS [29]. The integration of ML techniques with multi-omics approaches is expected to provide a comprehensive characterization of cardiovascular diseases, facilitate non-invasive assessments of AS, and potentially lead to the development of a “liquid biopsy” for cardiovascular applications [32]. Numerous bioinformatics tools are available for identifying co-expression networks, key driver genes, or differential networks. These include WGCNA (Weighted Correlation Network Analysis), Mergeomics, ARACNe (an algorithm designed for reconstructing gene regulatory networks within mammalian cellular contexts), and PANDA (which facilitates property transfer between networks for data assimilation). Multi-omics integration/trans-omics strategies amalgamate various omics modalities—such as genomics and transcriptomics; proteomics; and metabolomics—and analyze them concurrently. This approach aims to elucidate higher-order pathways involved in disease progression by examining complex molecular regulations governing phenotypes or cross-layer interactions [33]. The integration of omics data enhances our understanding of pathological mechanisms [26]. The advancement of network analytics and machine learning algorithms, when applied alongside large-scale multi-omics platforms, holds significant promise for uncovering novel biological networks. These methodologies are invaluable for both biomedical research and precision medicine initiatives [34]. By leveraging these algorithms—WGCNA for module detection, Mergeomics for cross‑omics causal inference, ARACNe for directed TF-target mapping, and PANDA for motif‑guided network refinement-researchers can extract biologically meaningful, mechanistically interpretable signatures from high-dimensional multi-omics data, thereby advancing both basic understanding and precision-medicine strategies for AS.The summary of bioinformatics tools for multi-omics analysis of AS is presented in Table 1.

**Table 1:** Summary of bioinformatics tools for multi-omics analysis in AS research

Bioinformatics tool	Core algorithmic principle	Specific application & unique advantage in AS research	Reference
**WGCNA**	Constructs scale-free weighted gene co-expression networks, clusters genes into functional modules via hierarchical clustering, and identifies hub genes based on intramodule connectivity (kME).	Applicable to bulk/single-cell transcriptomic/proteomic data from human AS plaques, carotid endarterectomy specimens or aortic tissue; identifies disease-relevant modules whose hub genes correlate with plaque burden, necrotic core size and vulnerability, serving as high-confidence candidate therapeutic targets and biomarkers.	[35]
**Mergeomics**	Integrates GWAS summary statistics with eQTL/pQTL and other multi-omics data, prioritizes cross-omics causal genes and pathways via meta-analysis and Bayesian causal inference.	Integrates AS-related GWAS summary statistics with blood/vascular tissue eQTL/pQTL data to prioritize cross-omics causal genes and pathways (e.g., PCSK9, LDLR), resolve pleiotropic risk loci, and support translational target validation.	[36]
**ARACNe**	Infers directed transcriptional regulatory networks based on transcription factor-target gene mutual information, excludes indirect interactions via the data processing inequality (DPI) to reduce false positive edges.	Applied to scRNA-seq data from human AS plaques to reconstruct cell type-specific transcriptional regulatory networks, identify master regulator TFs, and elucidate mechanisms of smooth muscle cell (SMC) phenotypic switching, neointima formation and plaque calcification.	[37]
**PANDA**	Integrates transcription factor DNA binding motif databases with gene co-expression information, assigns probabilistic confidence scores to regulatory edges, and generates condition-specific regulatory networks.	IApplicable to multi-omics data from vascular endothelial cells or plaque tissue to parse AS-specific transcription factor activity programs correlated with endothelial dysfunction and early lesion progression, enabling early intervention target screening.	[38]

## Application of integrative multi-omics analysis in AS

### Defining the pathogenesis of AS

The pathogenesis of AS is highly complex, and a full elucidation of its underlying pathophysiological cascades remains an unresolved challenge in cardiovascular research [39]. Multi-omics integration approaches have emerged as a transformative toolkit to address this gap, enabling comprehensive molecular profiling of disease-relevant primary tissues and systematically uncovering the molecular mechanisms driving AS progression [31].

Recent advances in this field have not been generated by isolated studies, but rather by a body of work that adopts differentiated multi-omics strategies aligned with distinct research questions, generating complementary and cross-referencing insights that collectively advance our understanding of AS pathogenesis. The integration of metagenomic and metaproteomic data has proven instrumental in identifying signaling proteins and pathways, revealing previously unknown proteins and signaling cascades implicated in the pathogenesis of AS [30]. Moreover, genetic testing combined with other omics disciplines, such as transcriptomics, can aid in the reclassification of variants of uncertain significance (VUS) and clarify their pathogenic mechanisms [40]. These two lines of work are inherently complementary: the former decodes the contributions of the microenvironment and microbiome to AS pathophysiology, while the latter bridges the gap between genetic variant detection and clinical risk stratification.Furthermore, the exponential expansion of cohort-derived and publicly available multi-omics datasets has enabled studies to move beyond pairwise integration to simultaneous profiling of multiple molecular layers. Studies that concurrently analyze RNA expression levels, protein concentrations, chromatin accessibility, and other disease-relevant molecular features are expected to illuminate fundamental biological processes and disease states associated with AS from a holistic integrative perspective [41]. Building on this progress, studies have adopted divergent integration strategies tailored to their specific research foci. For example, a systems biology study integrating genomic, transcriptomic, and metabolomic data from multiple independent cohorts (coupled with polysomnography, genetic analysis, RNA sequencing, and untargeted metabolomics) proposed that purinergic signaling mechanisms in sleep-disordered breathing (SDB) may drive the development of cardiovascular disease [42]. In contrast, a separate study integrating targeted whole serum lipidomics, targeted proteomics, and lipoprotein lipidomics focused specifically on dissecting the molecular basis of the association between metabolic syndrome (MetS, characterized by central obesity as a core diagnostic feature) and AS, by elucidating the mechanisms of lipoprotein remodeling [43].

Additionally, studies focusing on plaque-resident cell populations and therapeutic target identification have generated synergistic insights through the combination of multi-omics profiling and functional validation assays. A recent investigation combining proteomic and transcriptomic analyses of immune cells identified activated T cells and macrophages within atherosclerotic plaques [44], providing a direct molecular rationale for the development of SMC-targeted therapeutics. This finding is further corroborated and expanded by another study that combined fate mapping, lineage tracing, and single-cell RNA sequencing (scRNA-seq) to characterize the dynamic phenotypic landscape of smooth muscle cell (SMC) populations across all stages of AS [45]. More importantly,, a case-control study of human atrial tissue from cohorts with and without atrial fibrillation (AF) integrated genomics, transcriptomic, and proteomic data to dissect the shared and independent effects of cis-acting genetic variants on both transcriptional expression and protein abundance [46]. Collectively, these complementary multi‑omics investigations demonstrate that only through cross‑scale, cross‑phenotypic, and cross‑modal integration can the full mechanistic spectrum of AS be resolved, providing a robust molecular foundation for risk stratification and precision-targeted therapies.

### Facilitate early diagnosis and risk prediction of AS

The reversibility of AS lesions in early disease stages underscores the critical value of timely early diagnosis and risk prediction for disease reversal and targeted preventive intervention [47–48]. Current AS early diagnosis relies on three core dimensions: imaging techniques, gene/protein markers, and trace element detection, with the foundation of effective early screening lying in the integration of advanced diagnostic procedures and diverse examination methodologies [49]. Omics technologies, particularly multi-omics strategies encompassing genomics, transcriptomics, proteomics, and metabolomics, markedly accelerate biomarker discovery [50]. Elucidating the key processes involved in the transition from low-risk to high-risk rupture-prone plaques in humans will facilitate early diagnosis and targeted intervention for AS-related cardiovascular diseases. A comprehensive analysis of transcriptomic, proteomic, and peptidomic expression and abundance profiles of human carotid plaques can be employed to establish a multi-system prediction model that differentiates between low-risk and high-risk arterial lesions [51]. A study of 391 participants with early subclinical AS progression demonstrated that integrated analysis of whole-blood methylomics, transcriptomics, and plasma proteomics confirms epigenetic age as a personalized, accurate predictor of biological age [52].

Risk prediction serves as the cornerstone of personalized prevention strategies, aiming to direct interventions toward those individuals who are most likely to benefit [53]. Genetic scores, biomarkers, imaging studies, and multi-omics data all hold potential for identifying high-risk AS individuals [54], while early and rigorous control of risk factors can reduce AS incidence and slow disease progression, leading to long-term reductions in clinical adverse events [47]. Integrating diverse omics data significantly improves predictive accuracy, enhances causal inference capabilities, and clarifies underlying biological mechanisms [26]. Mediation analysis of transcriptomic and proteomic data has identified key pro-inflammatory pathways and genes mediating the link between subclinical AS and epigenetic age acceleration, highlighting the value of inflammatory interventions for cardiovascular disease prevention [52]. Advances in next-generation sequencing and computational tools now support pan-omics (corrected from original typo tuomics) exploration of interconnections across the genome, transcriptome, proteome and metabolome, overcoming limitations of traditional culture-dependent microbial research. Such integrated physiological phenotype analyses are expected to identify composite diagnostic/predictive biomarkers for risk stratification and precision medicine, and further improve AS risk prediction via integrative omics [55]. Noninvasive CAD omics models hold promise for identifying biomarkers worthy of further investigation, and are applicable for routine clinical use or pre-screening in target populations: individuals from low-risk Asian cohorts predicted to have subclinical AS (SA) are referred for ultrasound confirmation to access early preventive lifestyle modifications [56]. Whole-blood transcriptional profiles have also been validated as predictors of plaque characteristics [57]. Multi-level omics data integration enables mapping of complex biological networks in specific cells, tissues, individuals or populations to identify novel molecular targets, and combined with advances in statistical and algorithmic modeling, delivers unprecedented predictive performance [58].

### Explore new therapeutic targets and methods for AS

Despite decades of advances in cardiovascular disease (CVD) diagnosis and management, these conditions remain a major global health burden, with substantial unmet need for disease-modifying interventions beyond symptomatic relief [59]. Multi-omics integration, which reconstructs disease-relevant biochemical networks by interrogating interconnected metabolic, transcriptional, kinase-substrate, protein-protein interaction (PPI), and allosteric regulatory processes, offers a framework to address this gap [60]. Large-scale cohorts characterized by multi-omics signatures and robust clinical phenotyping facilitate novel mechanism and biomarker discovery, while next-generation genomic methodologies are required to resolve disease complexity and identify key progression drivers [61]. A more direct integration of genomics into drug development involves a relatively straightforward application of pharmacogenomics, where specific genetic variants are known to influence metabolism or modulate responses to therapeutic agents [62].

Two recent multi-omics lines of work illustrate complementary approaches to AS therapeutic target identification. One study employed an integrated approach that combined genetic, transcriptomic, and proteomic data from three vascular biobanks—encompassing carotid AS, thoracic aneurysms, and abdominal aneurysms—alongside clinical patient parameters and immunohistochemical analyses of vascular biopsies. This comprehensive analysis identified PCSK6, PCSK5, PCSK7, and FURIN as the proprotein convertases with the highest therapeutic potential [63], by contrast, single-cell sequencing of human atherosclerotic plaques to map gene regulatory network (GRN) drivers in female coronary artery disease (CAD) localized these active regulatory nodes predominantly to smooth muscle cells (SMCs), linking observed AS sex differences to SMC phenotypic switching [64]. Other multi-omics efforts focus on distinct translational axes: work centered on the gut microbiota-trimethylamine-N-oxide (TMAO) axis used assessments of microbial diversity, metagenomics and lipidomics to demonstrate that gypenoside effectively reduces plasma TMAO levels by remodeling gut flora and interfering with tricarboxylic acid (TCA) cycle and lipid metabolism-associated enzymes, subsequently modulating both TMAO and lipid metabolic pathways [65]; by comparison, studies evaluating blood-activating traditional Chinese medicine (BAC) botanicals in combination with antiplatelet agents leverage proteomic, lipidomic and miRNA analyses to characterize their anti-atherosclerotic targets and mechanisms in a clinically actionable context [66]. These mechanism-focused works dovetail with broader efforts combining metagenomics with genomics, transcriptomics, proteomics and metabolomics to develop targeted AS therapeutic strategies [67]. Table 2 presents the contributions of combined multi-omics analysis within the context of AS.

**Table 2:** The role of multi-omics joint analysis in AS

Multi-omics approach	Application in AS	Objectives	Mechanism	Limitations	References
**Genomics + metabolomics**	To investigate the synergistic effects of genetic variants and metabolic disorders.	To elucidate the mechanisms underlying the interactions between genetic risk factors and metabolic abnormalities.	PCSK9 gene mutations and the accumulation of oxidized lipids (ox-LDL) work synergistically to enhance plaque inflammation. The combination of PCSK9 inhibitors with lipid modulation therapy significantly reduces plaque burden.	Relies on single variant-metabolite associations, with unresolved polygenic-multi-metabolite interactions and limited generalizability from heterogeneous cohorts.	[68]
**Transcriptomics + proteomics**	To analyze the protein network regulated by non-coding RNA.	To reveal the molecular mechanism of lncRNA regulating macrophage function.	lncRNA MAARS modulates macrophage apoptosis and efferocytosis through its interaction with the HuR protein. The knockdown of MAARS leads to a reduction in macrophage apoptosis, an enhancement of efferocytosis, and an inhibition of plaque progression.	Bulk sequencing fails to distinguish macrophage subsets, with low transcriptome-proteome concordance and no defined clinical translation pathways.	[69]
**Microbiome + metabolomics**	To investigate the effect of gut microbiota metabolites on AS.	Developing microbiota modulation therapies.	Berberine addresses AS through a vitamin-like mechanism that downregulates the Choline-TMA-TMAO production pathway in gut microbiota.	Confounded by diet/antibiotic use, the causal direction of the choline-TMA-TMAO axis remains unclear, and no stable universal biomarkers exist.	[70]
**Radiomics + proteomics**	To develop a plaque prediction model based on imaging features	To improve the prediction accuracy of plaque rupture risk.	The plaque heterogeneity index (radiomics) and inflammatory proteins were integrated to develop a machine learning model aimed at predicting the risk of plaque rupture.	Poor radiomic feature reproducibility, small single-center cohort basis, and lack of large prospective clinical validation.	[71]

## Discussion

AS features complex, multifactorial progression mechanisms [72].Standardized large-sample multi-center studies integrating multi-omics approaches drive major advances in AS research [73]. Combining proteomics with transcriptomics, metabolomics and physiological phenotyping provides a robust framework to decode disease pathological features [74], while multi-omics analysis is increasingly adopted in biomedical research to map cross-molecular layer interactions. Multi-omics data underpins improvements in disease prevention, early detection, risk prediction, progression monitoring, phenotype classification, and development of personalized therapeutic strategies, including novel drug target discovery and biomarker identification [75]; public multi-omics datasets also facilitate cross-level analysis of risk factor-disease associations [31].

Key barriers limit clinical translation of multi-omics for AS. First, as an emerging field, multi-omics findings require rigorous validation before integration into AS clinical management, and overreliance on these techniques must be avoided when applying multi-omics and precision medicine frameworks to CVD research [76]. Second, translating integrated multi-omics data into actionable functional insights remains the field’s most critical bottleneck, necessitating development and optimization of dedicated multi-omics data integration and analysis tools [74]. Third, incorporating multi-species (human, mouse, porcine) and in vitro (2D/3D, static/shear stress) multi-omics data enables more accurate selection of pre-clinical models that recapitulate human disease global phenotypes or specific pathogenic mechanisms [33].

These three translational barriers delineate the critical unmet needs in atherosclerotic multi-omics research; bridging mechanistic discoveries to clinical application necessitates resolving interdependent methodological and operational hurdles across the entire analytical workflow. Multi-omics integration markedly improves our mechanistic insight into atherosclerotic disease by linking genetic variation, transcriptional regulation, protein abundance, metabolite flux and physiological phenotypes across cellular compartments. However, translating these gains into clinical practice remains hampered by several methodological and practical constraints.First, the high dimensionality of combined omics matrices amplifies over-fitting risks, especially when sample sizes are modest or derived from heterogeneous cohorts (different ages, ethnicities, comorbidities, sample‑collection protocols). Robust cross-validation, penalised regression or Bayesian hierarchical models are essential to mitigate spurious associations.Second, distinguishing causal drivers from correlative signatures persists as a core challenge. Even with Mendelian‑randomisation‑based approaches (e.g., Mergeomics), residual confounding, pleiotropy and tissue‑specific eQTL/pQTL effects can obscure true pathophysiological pathways; orthogonal validation in perturbation‑based models (CRISPR screens, organ‑on‑chip) is therefore required.Third, interpretability of complex machine‑learning classifiers (deep nets, ensemble methods) often limits biological insight. Incorporating explainable‑AI techniques (SHAP values, attention maps) alongside network‑centric analyses (WGCNA, ARACNe, PANDA) enhances mechanistic transparency.Fourth, multi‑omics studies entail substantial financial and logistical burdens-sequencing, proteomic metabolomic assays, stringent QC, and secure data‑storage infrastructure-restricting widespread adoption and limiting generalisability to under‑represented populations.Finally, successful translation demands rigorous functional validation of omics-prioritised targets in relevant in-vitro (2D/3D endothelial-SMC-macrophage co-cultures under physiological shear) and in-vivo (humanised mouse, porcine) models that recapitulate lesion phenotype. Development of open-source, reproducible pipelines for data harmonisation, batch-effect correction (e.g., ComBat-Seq, RUV) and multi-species mapping will be pivotal to overcome these hurdles and realise the promise of precision atherosclerosis medicine.

## Supplementary Information

Supplementary Material 1.

## Data Availability

No datasets were generated or analysed during the current study.
