# Supplementary material for: Application of multi-omics analysis in atherosclerosis
Source: J Cardiothorac Surg. 2026 Jul 29;21:845. doi: 10.1186/s13019-026-04648-y (PMC13700202; doi:10.1186/s13019-026-04648-y)
Supplement: Supplementary file 1 [file 13019_2026_4648_MOESM1_ESM.docx]

Supplementary Table 1 The Role of Single Omics in Atherosclerosis

| **Omics Type** | **Definition** | **Action Object** | **Study Objectives** | **Mechanism** | **Applications** |
| --- | --- | --- | --- | --- | --- |
| **Genomics** | Investigates genetic polymorphisms and other structural genetic variations | Genetic variations and single nucleotide polymorphisms (SNPs) | To identify the genetic risk factors associated with AS. | Mutations in the PCSK9 gene lead to increased LDL levels in the blood by interfering with LDL receptor recycling. | PCSK9 inhibitors significantly reduce LDL levels and become an important target for the treatment of AS. |
| **Transcriptomics** | Examines RNA expression patterns | mRNA, non-coding RNA (e.g., lncRNA, miRNA). | To analyze the influence of gene expression regulatory networks on AS. | Long non-coding RNA MIAT has been shown to upregulate the expression of CD47 and inhibit macrophage efferocytosis by acting as a sponge for miR-149-5p. | Knockdown of MIAT can enhance plaque stability and offers a novel strategy for the treatment of advanced AS. |
| **Proteomics** | Quantifies protein abundance | Protein expression and modification (e.g., oxidation, phosphorylation). | To identify biomarkers and drug targets associated with AS. | GDF-15 and TMAO, both inflammation-related proteins, contribute to plaque inflammation by activating the NF-κB signaling pathway. | The plasma level of GDF-15 is positively correlated with plaque instability, making it a valuable indicator for risk stratification in AS. |
| **Epigenomics** | Focuses on DNA methylation, histone modifications, lncRNAs and telomere length | DNA methylation, histone modification. | To reveal the effect of epigenetic regulation on AS plaque stability. | DNA methylation exacerbates intra-plaque inflammation by silencing anti-inflammatory genes such as IL-10. | Methylation inhibitors restore anti-inflammatory gene expression and improve plaque stability. |
| **Microbiome** | Assesses metabolic profiles | Gut microbiota and their metabolites (e.g., TMAO). | To explore the role of microbiota-host interaction in atherosclerosis. | TMAO promotes lipid accumulation and inflammation by inhibiting cholesterol reversal transport (RCT) and activating NLRP3 inflammasome. | Modulating gut microbiota to reduce TMAO levels reduces plaque burden (Phase II clinical trial). |
| **Metabolomics** | Analyzes the composition and taxonomic classification of the gut microbiome | Metabolites (e.g., ox-LDL, bile acids). | To analyzed the relationship between metabolic disorders and the progression of AS . | ox-LDL activates the inflammatory response of macrophages through LOX-1 receptor and promotes foam cell formation. | Antibody drugs targeting LOX-1 can reduce plaque lipid core formation and reduce the risk of plaque rupture. |
